# Hybrid debranching and TEVAR of the aortic arch off-pump, in re-do patients with complicated chronic type-A aortic dissections: a critical report

**DOI:** 10.1186/1749-8090-8-188

**Published:** 2013-09-04

**Authors:** Klaus Brechtel, Günay Kalender, Ulrich A Stock, Stephen M Wildhirt

**Affiliations:** 1 Department of Radiology, University Hospital, Tübingen, Germany; 2 Department of Vascular Surgery, Hoechst Hospital, Frankfurt, Germany; 3 Department of Cardiac- and Vascular Surgery, University Hospital, Frankfurt, Germany; 4 Isar Heart Center, Munich, Germany

**Keywords:** Aortic debranching, Off-pump surgery, TEVAR, Aortic dissection

## Abstract

**Background:**

Patients suffering from acute type A aortic dissection undergo replacement of the ascending aorta, the proximal hemiarch or complete aortic arch, depending on the extent of the individual pathology. In a subset of these treated patients, secondary pathologies of the distal anastomosis or the remaining distal part of the aorta occur. The treatment of these pathologies is challenging, requiring major surgical re-do procedures with aortic arch replacement under extracorporeal circulation and hypothermic circulatory arrest.

**Methods:**

We report our experience of five patients with complex aortic pathologies after previous aortic surgery treated with a single stage re-do hybrid procedure, consisting of bypass grafting of the supraaortic branches off-pump, stent graft placement for endovascular aortic repair (TEVAR) and surgical debranching of the aortic arch.

**Results:**

In all patients the surgical vascular grafts and stent grafts were deployed successfully, there were no intraoperative deaths. Four out of five patients were discharged from hospital in good clinical condition. One patient died postoperatively due to cardiac tamponade. In one patient a type I endoleak persisted leading to occlusion of a bypass branch requiring surgical revision at one year after debranching.

**Conclusion:**

We discuss the prerequisites, all steps and potential pitfalls of this hybrid aortic arch replacement. The current procedure avoids cardiopulmonary bypass and circulatory arrest, which may benefit early patient outcome; however, patient and device selection plays a key role for immediate success and midterm outcomes. In addition, precise procedural planning and development of customized stents may help to develop this procedure into a true alternative for conventional aortic arch replacement.

## Background

Acute aortic dissection type A (AADA) remains a challenging and often fatal disease. Patients are mostly treated under emergency conditions. Depending of the extent of the injury, surgery consists of resection of the aortic tear and replacement of the ascending aorta, the hemiarch or arch replacement using extracorporeal circulation and hypothermia with or without circulatory arrest. The surgical procedure has a favorable mid- and long-term prognosis of approximately 90% survival at one year, 72-77% at five years, and 53-56% at ten years [1-4]. However, life-long follow-up of these patients is manadatory because they are prone to procedure- or dissection-related short-, mid- and long term complications. Indeed, most of the patients suffer from hypertension, a major risk factor that often remains insufficiently treated post-surgery.

Typical procedure related complications are pseudoaneurysms or development of true aneurysms at the proximal or distal sutureline and re-dissections. These problems have been repeatedly reported with the use of various surgical techniques including the use of glue or Teflon-felt reinforcement of the suture lines [5,6]. Although these pathologies may occur at both, the proximal and/or distal anastomosis of aortic replacement, the present report focuses on distal aortic pathologies.

Following aortic surgical procedures, aneurysmatic dilatation of the residual aorta with the subsequent risk of rupture remains the main problem long-term. An important goal in resecting the (all) aortic tear(s) is the occlusion of the false lumen distal to the repair. However, in the majority of patients a chronically dissected false lumen within the distal aorta remains perfused [1,3]. As long as there is no clinical sign for organ malperfusion, extravasation of blood or signs for progression of dilation/dissection no further intervention may be required. However, the treatment of these secondary pathologies is challenging, requiring major surgical intervention (aortic arch replacement, i.e. ‘frozen elephant trunk procedure’) with hypothermic circulatory arrest and selective antegrade cerebral perfusion. As redo procedures these interventions are associated with a high risk of bleeding, cerebral ischemia/stroke, visceral malperfusion and death.

The procedure and device development for thoracic endovascular aortic repair (TEVAR) led to the development of hybrid procedures for aortic (arch) repair in the last decade. Their common concept is bypass grafting of the supraaortic branches and ligation of the native branches (“debranching”) followed by endovascular stent grafting of the aortic arch with or without subsequent TEVAR of the descending aorta. The term “partial debranching” refers to operations with revascularization of just the left carotid artery and subclavian artery, i.e. by carotid-carotid bypass whereas “total debranching” also includes revascularization of the brachiocephalic trunk.

We adopted the concept of hybrid aortic arch repair for the subpopulation of patients with chronic dissections after ascending aortic- and hemiarch replacement with significant pathologies of the remnant distal aorta and with an excessive risk for conventional repair. To our knowledge this is the first series specifically dealing with this particular subset of patients treated with single-stage hybrid arch repair off-pump.

## Methods

### Patients’ enrollment

Annually, approximately 30 patients with AADA are referred to our institution for emergent surgery. Postoperatively, all patients were enrolled in a follow-up program including clinical examination and imaging studies to assess changes in the chronical disease process. In August 2010 we introduced the strategy of hybrid aortic arch repair consisting of debranching and endovascular stent grafting described herein as a complementary strategy of our conventional redo aortic arch surgery program.

All patients requiring redo arch procedures were discussed in a multidisciplinary board to decide for the appropriate therapy. Prior to surgery all patients underwent computed tomography (CT) scan, coronary angiography, echocardiography and duplexsonography of the carotid arteries. Both procedures, conventional redo aortic arch replacement and hybrid aortic arch repair were discussed with the patients. Based on the individual risk profile (age, secondary organ dysfunction, vascular access site as well as technical and anatomical considerations regarding stent placement) the multidisciplinary board recommended the one or the other. Between August 2010 and April 2011 five patients were enrolled for redo arch procedures applying the hybrid procedure. All patients were informed about their complex disease process and the none-standardized nature of the surgical procedure and gave their written informed consent. All procedures and techniques applied are in accordance and compliance with the principles of the Declaration of Helsinki.

### Anesthesia and monitoring of the patients

All procedures were performed under general anesthesia with Sufentanil, Sevoflurane and Recuronium after induction with Midazolam. Standard cardio-circulatory monitoring with an arterial line in the right radial artery, and central venous- and pulmonary artery catheters was supplemented by transesophageal echocardiography. During the entire procedure near-infrared spectroscopy (NIRS) was used for neuromonitoring.

### Surgical procedure: aorto-bicarotid bypass grafting and aortic debranching

**Figure 1:**
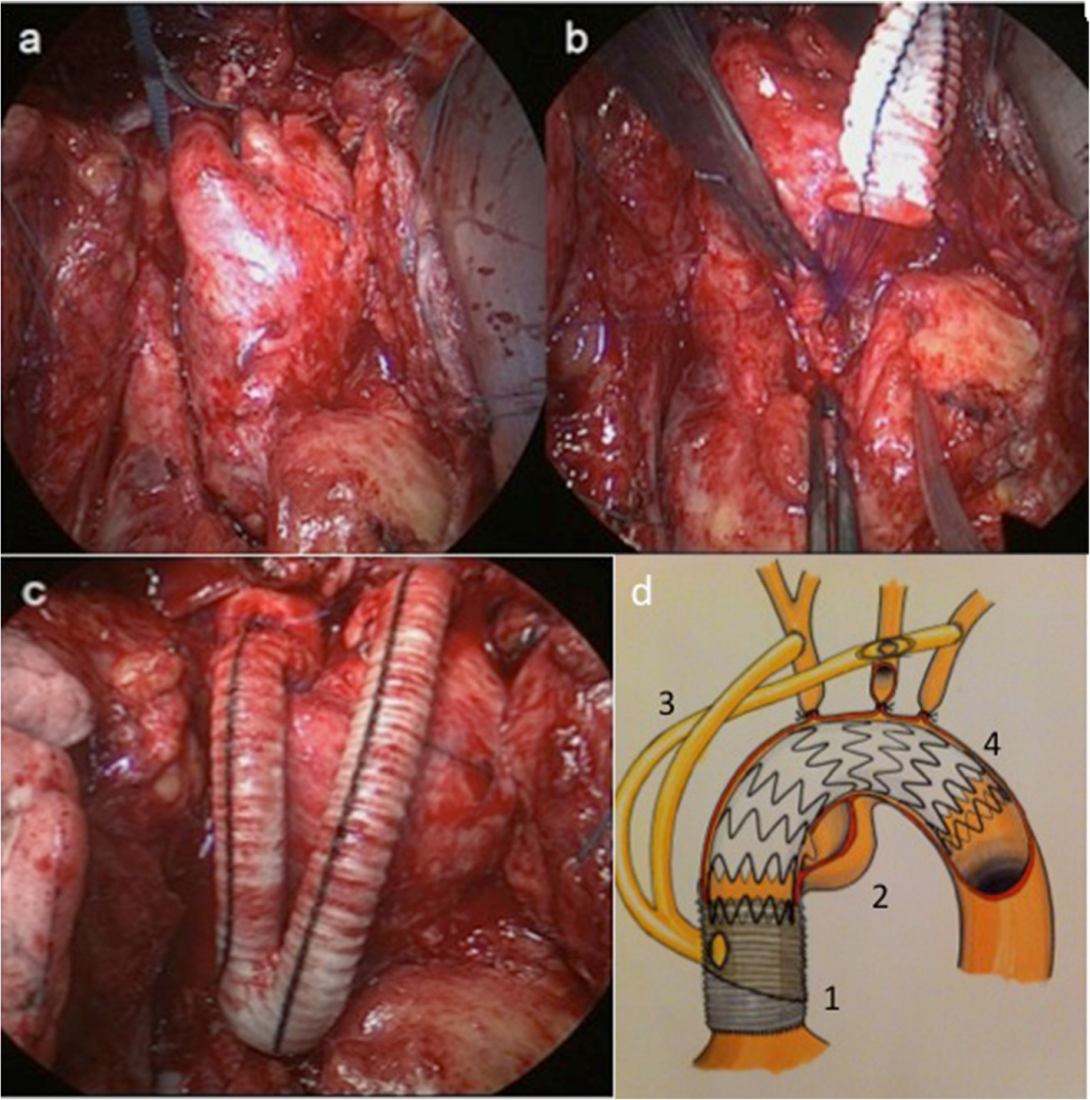
**Surgical dissection, Y-graft placement and positioning.** Surgical procedure: after redo sternotomy, the ascending aorta and the aortic arch with its branches are dissected **(a).** As proximal as possible, a Y-shaped prostheses is anastomosed end-to-side at the ascending aorta prostheses **(b)**. Distal anastomoses to the brachiocephalic trunk (end-to-side), the left carotid artery (side-to-side) and the left subclavian artery (end-to-side) (**c**; patient #2). Cartoon showing the surgical anastomosis to the aortic prosthesis **(d)**; 1: Aortic Prosthesis from previous surgery, 2: New, secondary aortic pathology (i.e. aneurysm, re-dissection), 3: Y-graft, 4: Stentgraft.

The chest was opened with redo sternotomy with cardio-pulmonary bypass (CPB) standby. In two patients (#s 1 and 3) CPB was initiated femo-femoral during sternotomy only to empty the heart but was terminated immediately after opening of the chest. The remaining procedures were performed off-pump in all cases. The aortic structures including supraaortic vessels and the right aspect of the heart were dissected. Care was taken to dissect the aortic prosthesis as much as possible beyond the proximal suture line. Epicardial pacemaker wires were placed in standard fashion. Heparin (5000 IE) was administered systemically. An Y-shaped Dacron® prosthesis (Uni-Graft®, Braun, Melsungen, Germany) was chosen (Table 1). With a side biting clamp placed as proximal as possible on the ascending aortic prosthesis the first anastomosis was performed end-to-side. Next the brachiocephalic trunk (end-to-side), the left carotid artery (side-to-side) and the left subclavian artery (end-to-side) were anastomosed to the smaller branches of the prostheses using side biting clamps to ensure residual perfusion (Figure 1). Intraoperatve angiography was employed to assess the quality of the anastomoses. The native supraaortic vessels were left open until completion of the stent placement (see below). After successful placement of the stent graft was confirmed by angiographic control, the supraaortic vessels were ligated. After placement of chest tubes and haemosthasis, the chest was closed in standard manner and patients were transferred to the intensive care unit.

**Table 1:** Prosthetic material used for aortic reconstructions during the different stages of surgery

***Patient***	***Prostheses for ascending aorta replacement***	***Prostheses for revascularization of the supraaortic branches***	***Endovascular stent graft prostheses for aortic arch reconstruction***
#1	30 mm straight Dacron tube graft	16/8/8 mm Y-shaped-Dacron-Prosthesis	Valiant Captiva*; 36; 36; 200
#2	26 mm straight Dacron tube graft	14/7/7 mm Y-shaped-Dacron-Prosthesis	Valiant Captiva*; 34; 34; 200
#3	30 mm straight Dacron tube graft	14/7/7 mm Y-shaped-Dacron-Prosthesis	Relay Plus (tapered custom made device)**; 32; 28; 155
#4	30 mm straight Dacron tube graft	16/8/8 mm Y-shaped-Dacron-Prosthesis	Valiant Captiva*; 34; 34; 100
#5	26 mm straight Dacron tube graft and 21 mm biological aortic valve prosthesis	14/7/7 mm Y-shaped-Dacron-Prosthesis	Valiant Captiva*; 32; 32; 150

(*) Medtronic (**) Bolton Medical. Stent grafts: brand/type; proximal diameter (mm); distal diameter (mm); length (mm).

### Transfemoral endovascular stent grafting of the aortic arch

**Figure 2:**
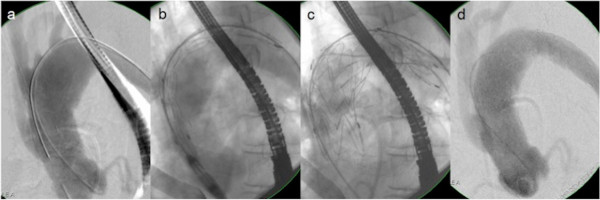
**Fluoroscopy for Y-graft identification, stent positioning/delivery and post-Op control.** Interventional placement of the endovascular prosthesis (transfemoral approach): Angiography of the ascending aorta, aorto-bicarotid bypass, aortic arch and supraaortic vessels **(a)**. Positioning of the delivery-catheter and the endovascular prosthesis **(b)**. Unfolding and final placement of the endovascular prosthesis **(c)**. Angiography after placement of the endovascular prosthesis and debranching of the aortic arch **(d)**. (Patient #3).

Pre-interventional CT-scan was performed using ultrafast multi detector CT (Somatom Definition FLASH, Siemens, Erlangen, Germany) using contrast media (Imeron 400, Bracco, Konstanz, Germany). For interventional procedure planning measurements were performed from axially reconstructed 1 mm arterial phase CT images (SyngoVia, Siemens, Erlangen, Germany). Centerline measurements were used for length detection. Orthogonal diameters according to centerline measurements were applied for diameter sizing. Sizing priority was focused on the proximal sealing zone within the ascending aorta. Oversizing of the stent graft was about 10-20% with respect to the proximal diameter. Overstenting of the aorto-bicarotid bypass with the proximal bare stents was considered acceptable as long as the fabric did not cover the bypass anastomosis and the landing zone was at least 10 mm according to pre-interventional CT. However, the origin of the aorto-bicarotid bypass could not always be predicted and required reevaluation during the intervention. Strong aortic kink or angled course of the ascending aorta was accepted as relatively contraindication as long as the stent graft delivery system reached the ascending aorta and the proximal cone passed the aortic valve. Stent grafts chosen for each individual patient are specified in Table 1. Three stent grafts were standard off-the-shelf devices (pts. #1, #2, and #5), one stent graft was customized within two weeks prior to the procedure (pt. #3), and one stent graft was customized during the procedure by shortening the distal part by 20 mm. Stent placement was performed under fluoroscopy guidance (Arcadis Varic, Siemens, Erlangen, Germany). One common femoral artery was punctured and a 5 french sheath (Radiofocus, Terumo; Leuven, Belgium) was inserted. A calibrated angiography catheter (Performa, Merit Medical, Eschborn, Germany) was placed in the ascending aorta and the aortic arch. The contralateral common femoral artery was approached via surgical cut down. After placing a 11 F sheath (Radiofocus, Terumo, Leuven, Belgium) a superstiff wire was advanced into the aortic arch. The tip of the wire passed through the aortic valve and was placed into the left ventricle. The stent graft was retrogradely advanced into the desired position. Before stent graft deployment, rapid pacing via epicardial pacemaker wires (160-180 bpm) was initiated to obtain a short period of tachycardiac low output. After final correction of the position, the stent graft was deployed. Figure 2 illustrates the process of delivery of the endovascular prosthesis.

Stent graft placement was followed by angiographic assessment for endoleaks and Y-graft patency. In case of type Ia endoleak post-dilatation of the landing zone was performed using a compliant balloon (Reliant, Medtronic, Meersbusch, Germany). Type II endoleak was corrected surgically by occlusion of the native supra-aortic vessels. Low flow endoleaks were accepted in order to monitor the hemodynamic effect.

## Results

### Patient demographics, preoperative status and indications for redo surgery

Between August 2010 and April 2011, five patients were identified with a significant pathology of the distal aortic anastomosis. Patient demographics are depicted in Table 2.

**Table 2:** Patients’demographics and accompanying diseases

***Patient***	***Age at debranching (years)***	***Sex***	***Accompanying diseases***
#1	73.08	M	AHT, HLP, CHD, COLD, DM
#2	79.09	F	AHT; HLP; CHD; RA
#3	81.99	F	AHT; RA
#4	70.99	F	AHT
#5	70.83	F	AHT, HLP, DM, CAD

*AHT* Arterial hypertension, *HLP* Hyperlipidemia, *CHD* Coronary heart disease, *CAD* Carotid artery disease, *COLD* Chronic obstructive lung disease, *DM* Diabetes mellitus, *RA* Rheumatoid arthritis.

Each patient presented with a complex individual disease progression. In all patients the proximal aortic anastomosis and valvular function was intact.

**Figure 3:**
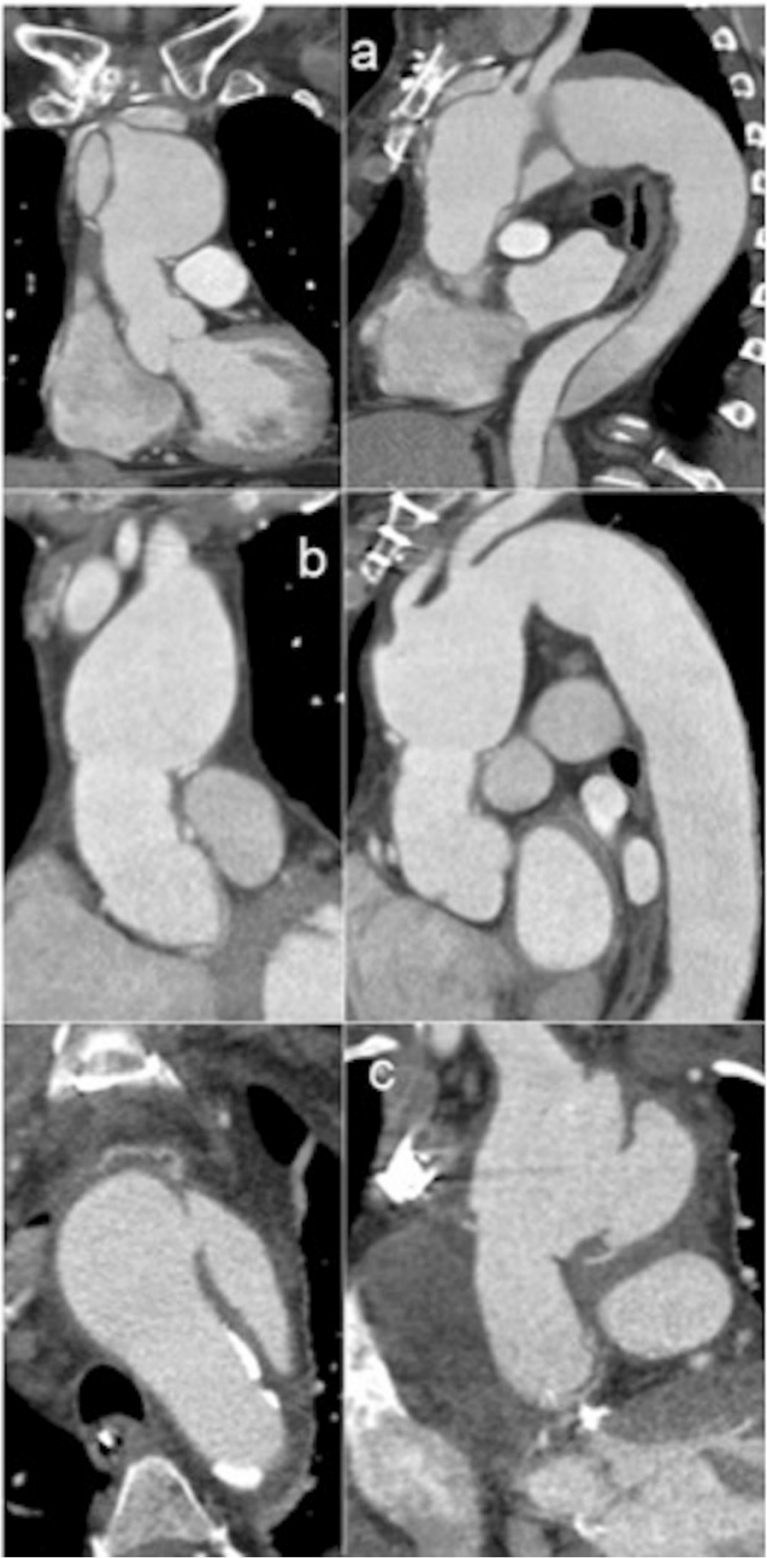
**Various pathologies of distal aortic anastomosis.** Distal anastomosis’ pathologies: Dissection and consecutive dilatation of the aortic arch (**a**; Patient #1). True aneurysm of the aortic arch (**b**; Patient #2). Pseudoaneurysm of the distal anastomosis, posterolaterally (**c**; Patient #5).

In two patients (#1 and #3), the dissected arch had developed into an aneurysm. In patient #1, the arch had enlarged to a maximum diameter of 7.1 cm with alteration of the recurrent laryngeal nerve (Figure 3a) six months after replacement of the ascending aorta and hemiarch. In patient #3 surgery for AADA was performed more than seven years before. In patient (# 2), the aortic arch showed a significant true aneurysm formation (Figure 3b). In the two remaining patients, formation of a pseudoaneurym at the distal aortic anastomosis was found (#s 4 and 5) (Figure 3c). Time point of occurrence of pseudoaneurysm was within 4 weeks following initial surgery (#5) and more than 8 years after initial surgery (#4). Detailed descriptions of the primary aortic pathologies and initial surgical procedures performed as well as the secondary aortic pathologies which developed subsequently are presented in Table 3.

**Table 3:** Primary surgery at the time point of acute aortic dissection, time period between initial surgery and redo procedure, pathology at time-point of redo surgery

***Patient***	***Primary surgery***	***Years between primary and redo surgery***	***Pathology prior to debranching***
#1	Supracomissural ascending aorta replacement, hemiarch replacement	0.52	Chronically dissected aortic arch, consecutive dilatation
#2	Supracomissural ascending aorta replacement, open distal anastomosis	4.35	Progressive aneurysm of the arch and descending aorta
#3	Supracomissural ascending aorta replacement, open distal anastomosis	7.18	Chronically dissected aortic arch, consecutive dilatation and true lumen collaps
#4	Supracomissural ascending aorta replacement, open distal anastomosis	8.69	Pseudoaneurysm of the distal anastomosis
#5	Aortic valve- and root replacement (conduit), hemiarch replacement	0.11	Pseudoaneurysm of the distal anastomosis

### Intraoperative results

In all patients simultaneous surgical and interventional procedures were performed successfully. In two patients (#s 1 and 3) short periods of CPB where required for safety reasons during redo sternotomy because of severe adhesion of the heart to the posterior aspects of the sternum. The complete bypass- and debranching procedures were performed off-pump.

In all patients bypass grafting between the preexisting ascending aortic prosthesis and both supraaortic arteries and stent graft placement was achieved without complications. All patients returned to spontaneous sinus rhythm with stabke hemodynamics after terminating rapid pacing.

**Figure 4:**
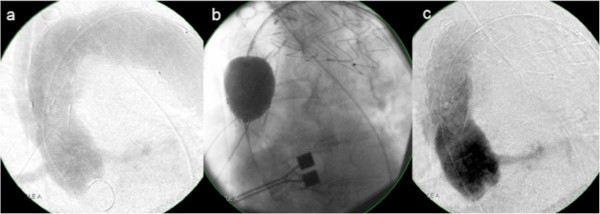
**Successful re-ballooning of Type-1 endoleak.** Intraoperative closure of an endoleak type I **(a)**, immediate therapy by reballooning **(b)**, postinterventional result **(c)** (Patient #5).

**Figure 5:**
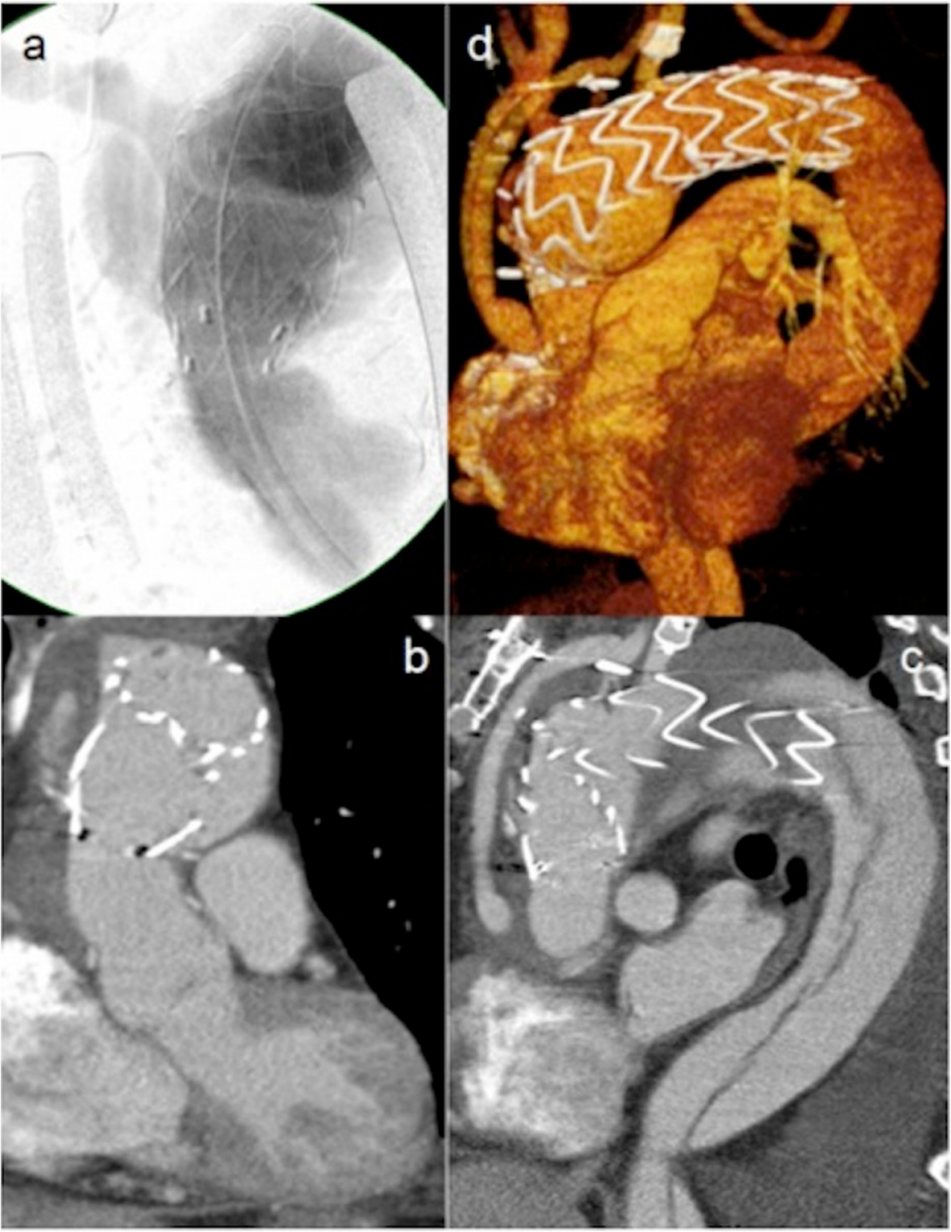
**Persistance of Type-1 endoleak.** Significant type I endoleak in Patient #1: intraoperative angiography **(a)**, frontal- **(b)**, sagital **(c)** and 3-dimensional **(d)** reconstructions postoperatively.

Intraoperative angiographic control revealed type 1a endoleaks in two patients (#s 1 and 5). In patient #5 the endoleak was successfully treated by reballooning of the proximal aspects of the stent graft (Figure 4). However, in patient #1 reballooning failed. The stent graft prostheses buckled at the inner curvature of the preexisting hemiarch replacement. The resulting wrinkles caused a migration of the stent graft distally, resulting in the persistence of a typ Ia endoleak despite repeat reballooning (Figure 5). The endoleak persisted at 3 and 6 months follow-up. Because of the risk of further progression the patient was informed and treated surgically with an elephant-trunk (Table 4).

**Table 4:** Intraoperative and postoperative times, postoperative complications and results of the mid term follow-up

***Patient***	***Operation time (min)***	***CPB time (min)***	***ICU time (days)***	***Hospital stay (days)***	***Perioperative period***	***Chronic postoperative period***
#1	536	29	1	10	Uneventful	Persisting Type I Endoleak, occlusion of one bypass branch with consecutive cerebral malperfusion. Redo elephant trunk.
#2	386	0	3	13	Uneventful	Type II endoleak via the brachiocephalic trunk, insignificant.
#3	386	38	1	1	Early postoperative death due to cardiac tamponade	-
#4	355	0	1	10	Uneventful	Uneventful
#5	415	0	23	23	Delayed: Pneumonia, delirium, critical illness polyneuropathia, previous cerebral infarction	Died six months postoperativey due to pulmonary embolism

All patients survived the procedure and where transferred to the intensive care unit on low dose catecholamines.

### Perioperative results

One patient, #3, unexpectedly died fife hours postoperatively. During weaning from the respirator, therapy-refractory ventricular fibrillation occurred. After mechanical resuscitation for 10 minutes, massive intrathoracic bleeding occurred, most likely due to injury of the right ventricle (this patient had severe retrosternal adhesions requiring extensive dissection during surgery). There was no hint that the bleeding occurred primarily. The actual cause of her death however remains unclear as an autopsy was denied by the relatives.

All other patients survived the perioperative period. In Table 4 the durations of intensive care unit- and total hospital stays are depicted.

Patients #1, 2 and 4 experienced an uneventful early postoperative period, where transferred to the normal ward after a one day ICU-stay and where discharged from hospital 10 to 13 days after surgery in excellent general condition. In Patient #1, the endoleak persisted through the perioperative period without any symptoms with otherwise uneventfull clinical recovery.

Patient #5, who had undergone initial aortic repair with a biological valve conduit 4 weeks earlier had already presented with multiple cerebral infarctions after the first operation. After the debranching procedure, she developed a delirium and a viral pneumonia, both resulting in delayed weaning from respirator. However, no new signs for neurological impairment were found. The patient was referred to specialized neurological rehabilitation after successful weaning after 23 days.

### Short-term follow up

All surviving patients where seen in our outpatient -clinic three, six and twelve months after surgery.

Patients #2 and 4 had recovered properly. In Patient #2, a small and insignificant type II endoleak via the brachiocephalic trunk was evident despite ligation of the vessel. As this endoleak shrank significantly during the first six postoperative months and the diameter of the aneurysm was consistent it was judged as irrelevant. One year after hybrid aortic arch replacement, both patients presented in excellent clinical condition.

Patient #5 recovered initially with the preexisting left-sided hemiparesis and a slight dysphagia. However, she died six months after the aortic procedures due to pulmonary embolism, apparently unrelated to the debranching procedure. All bypass grafts in Patients #2, #4 and #5 were patent six months postoperatively.

In patient #1 weakness of the right arm was observed 3 months postoperatively. After referral to our institution on CT imaging, partial obstruction of the left sided branch of the Y graft was diagnosed treated with tromboembolectomy of the bypass. However, in the further period the bypass branch occluded, likely due to the persisting Type-1 endoleak. The patient underwent redo surgery with replacement of both, the aorto-bicarotid bypass and the endovascularprostheses and by a frozen elephant trunk (E-vita open, Jotec, Hechingen, Germany) eleven months after the debranching procedure. Recovery of the Patient was uneventful, he was discharged from hospital in excellent condition after 2 weeks.

### Outcome classification

According to the reporting standards for thoracic endovascular aortic repair of the society for vascular surgery [7], the outcome was classified as follows: *Severe* complication occurred in two patients, #1 with endoleak type I and branch occlusion of the aorto-bicarotid bypass, and patient #3 with early a perioperative death (likely to be classified as *TEVAR-related)*.

Table 5 summarizes patient’s outcome results. The postinterventional neurological impairment and prolonged need for intensive care in patient #5 was related to the first aortic surgery.

**Table 5:** Outcome according to the nomenclature of the reporting standards for TEVAR, Fillinger et al.

***Patient***	***Technical success***	***30 day-clinical success***	***Short term (6months) clinical success***	***Deaths***
#1	-	-	-	-
#2	+	+	+	-
#3	-	-	-	Early, TEVAR-related death
#4	+	+	+	-
#5	+	+	-	Late, TEVAR unrelated death

The insignificant endoleak type II in Patient #2 is only classified as a *mild* complication.

*Technical success* was not achieved in two out of five patients, due to the endoleak in patient #1 and the early perioperative death in patient #3.

In the remainig patients #s 2, 4 and 5 *technical*- and *clinical success* was achieved.

## Discussion

The need for re-operations for distal aortic pathologies after repair of aortic dissection may be around 25% within the first 5 years and even higher. In adition, re-operations bare a high risk for complication including stroke, prolenge bleeding and death (4).

We report our experience of five patients with complex aortic pathologies secondary to previous aortic surgery for type-A acute aortic dissection. We performed a single stage, re-do hybrid procedure off-pump, consisting of bypass grafting of the supraaortic branches, stent graft placement for thoracic endovascular aortic repair (TEVAR) and surgical debranching of the aortic arch.

To our knowledge this is the first series of re-do patients with this complex subset of aortic pathologies treated with single stage off-pump hybrid aortic arch repair.

The conceptional development of hybrid aortic arch replacement is of growing interest, particularly in patients with excessive risk for conventional redo aortic-arch surgery. While we focused on secondary pathologies after ascending aorta replacement for AADA, most series report the outcome of hybrid aortic arch repair for miscellaneous pathologies.

In 2010 Milewski et al. compared two cohorts undergoing either conventional or hybrid aortic arch replacement. They presented similar outcomes and concluded that debranching is a safe alternative for high risk and elderly patients (>75 years) [8]. Similarly, Lee at al. compared two cohorts undergoing either the elephant trunk procedure with endovascular completion or aortic debranching followed by endovascular arch replacement. The authors found similar outcomes of both procedures but reported reduced requirement for cardiopulmonary bypass in the debranchig group [9]. Ferrero et al. came to a similar conclusion by reviewing their series of 27 Patients with a 30-day mortality of 11.1% and just one Type I endoleak [10]. However, these series deal with multiple aortic arch pathologies such as true aneurysms, penetrating aortic ulcers and dissections. Marullo et al. described a series of 24 patients with DeBakey I dissections, which underwent urgent ascending aorta and –arch replacement with debranching as the initial procedure, 15 of whom in a second stage underwent endovascular stent graft implantation. He reported a low mortality of just one patient, a neurological dysfunction rate of just 12.6% and excellent mid-term results with respect to false lumen thrombosis [11].

These are encouraging results. However, others have reported more critical data: Geisbüsch et al. reported a series of 47 patients undergoing mostly staged complete or partial debranching. The authors found an overall in hospital mortality of 19%, even 27% among those patients with total debranching. Type I endoleaks appeared in almost 15% and the rate of neurological complications was 12%. They recommended hybrid arch procedures to be performed only in high volume centers [12].

Similar with Geisbüsch’s experience, we also saw relevant complications in our patients, but these may differ from the complications seen with conventional aortic surgery. For instance, intra- und prolonged postoperative bleeding is a major risk for conventional and staged hybrid arch replacement were extended cardiopulmonary bypass and hypothermia is required. This may be reduced when avoiding CPB, as described here. However, due to the risk of increased bleeding in redo procedures during sternotomy standby of extracorporeal circulation is mandatory. In fact, in two patients we performed resternotomy using preload reduction on CPB.

Cerebral ischemia due to unstable hemodynamics, hypoperfusion or (air) embolic events is another risk of the conventional procedure. In contrast, in the off-pump approach, we found very stable hemodynamics throughout the entire procedure, even under placement and removal of the side-biting clamp at the ascending aorta prosthesis. We performed neuromonitoring by NIRS in every patient (which in our opinion is mandatory) and saw no significant alterations during the procedure.

Branch obstruction may be the main risk for cerebral malperfusion in the chronic phase after hybrid aortic arch replacement, and may be caused by two important aspects. First, anatomic positioning of the branch directly behind the sternum predisposes to compression. Second, the increasing diameter of the false lumen of the native aorta through the Type-1 endoleak may have had an impact on branch compression. This clearly shows, that the success of this procedure is only given with the success of each individual step. In this particular patient, the angulation of the distal part of the previously implanted aortic prosthesis and the native dissected aorta was steep. Therefore, the landing zone for the stent graft was short and the angulation resulted in strong radial forces on the stent graft resulting in malposition and insufficient radial expansion. We believe that the occlusion of one bypass branch in patient #1 was due to these two factors. This complication also has been observed and described by others [12]. Consequently, compression of a bypass branch after sternal closure has to be carefully avoided and Type-1 endoleaks have to be treated early.

Optimal placement of the proximal end of the endovascular stent graft in the landing zone is the most critical point in the endovascular part of the intervention. Positioning to far proximally may impair perfusion of the proximal anastomosis of the aorto-bicarotid bypass or even the coronary arteries. Placement to far distally may result in dislocation of the prostheses and endoleak type I. Most authors recommend a landing zone of at least 1.5 to 2 cm to enable for secure delivery of the prosthesis. To avoid dislocation during deployment of the endovascular prosthesis, lowering cardiac out-put is highly recommended, in our case achieved by rapid pacing. Despite all these maneuvers, in two of our patients significant type I endoleaks were observed. In one patient it resulted from incomplete unfolding of the prostheses and was successfully treated by reballooning. In the second patient it resulted in incomplete radial expansion of the prosthesis due to steep angulation of the original arch and a short landing zone of less than 15 mm resulting after implantation of the aorto-bicarotid bypass.

With respect to long-term outcome patients require a thorough follow-up mostly by repeated contrast enhanced CT. Stabile stent positioning, possible progression of remaining aneurysm, secondary type I endoleaks have been described and may be of concern.

## Conclusions

The described procedure of single-stage aortic arch debranching after implantation of an aorto-bicarotid bypass and subsequent endovascular stent grafting represents an alternative to conventional redo aortic arch replacement and the frozen elephant trunk in patients with chronic AADA after ascending aorta replacement. In selected cases it can be performed completely off-pump. However, the described procedure remains a major surgical intervention with relevant risks. The main difference as compared to traditional surgical arch repair is the avoidance of circulatory arrest and hypothermia. The procedure requires precise interdisciplinary planning by an experienced team of cardiac and vascular surgeons, interventionalists and imaging specialists. The continued development of (customized) stent grafts will reduce the risks for endoleaks, ongoing perfusion of the aneurysm and stent migration. Criteria for patient selection should include the interpretation of the arch anatomy and angulation, precise measurement for placement of vascular grafts and landing zone determination.

Conventional arch surgery, even in redo patients may also be performed with acceptable risks. Given the development of improves cerebral protection during recent years and the excellent results achieved with conventional aortic arch replacement, it remains to be determined which patient will be the optimal candidate for one or the other procedure.

Based on our experience, we believe that this hybrid procedure represents an alternative treatment option in selected patients if conventional arch surgery is associated with an inappropriate risk. Future studies with precise, individualized procedure planning using customized stents may improve the hybrid off-pump technique to become a true alternative to the established surgical procedures.

## Abbreviations

AADA: Acute aortic dissection type A; TEVAR: Thoracic endovascular aortic repair; CT: Computed tomography; NIRS: Near-infrared spectroscopy; CPB: Cardio-pulmonary bypass; ICU: Intensive care unit; AHT: Arterial hypertension; HLP: Hyperlipidemia; CHD: Coronary heart disease; CAD: Carotid artery disease; COLD: Chronic obstructive lung disease; DM: Diabetes mellitus; RA: Rheumatoid arthritis.

## Competing interests

The authors declare that they have no competing interests.

## Authors’ contributions

KB planned and carried out the interventional radiology parts of the procedure, collected and analysed the data. GK, US, and SW planned and carried out the surgical parts of the procedure. SW initiated and designed the study, collected and analysed the data and wrote the manuscript. All authors read and approved the final manuscript.

## Authors’ information

The study was undertaken at the University Hospital of Tübingen. Currently, GK, UA and SW work in other institutions.
